# Supplementary figures and images for: Divergent features of the coenzyme Q:cytochrome c oxidoreductase complex in Toxoplasma gondii parasites
Source: PLoS Pathog. 2021 Feb 1;17(2):e1009211. doi: 10.1371/journal.ppat.1009211 (PMC7877769; doi:10.1371/journal.ppat.1009211)

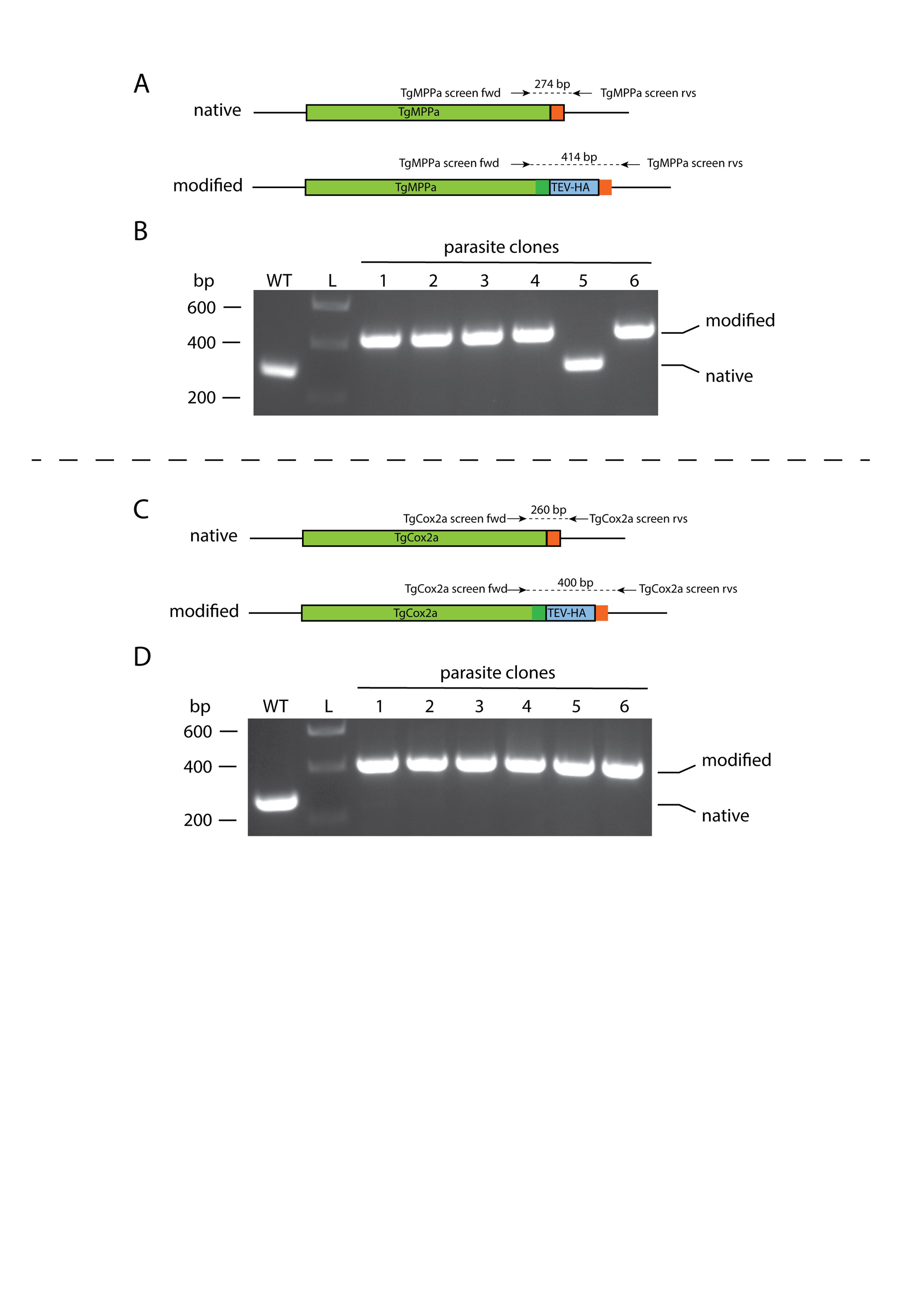

Supplement: S1 Fig — (A) Diagram depicting the 3’ replacement strategy to generate TEV-HA-tagged TgMPPα. A sgRNA was designed to target the T. gondii genome near the stop codon of TgMPPα, and cause a double stranded break. A plasmid containing the sgRNA and GFP-tagged Cas9 endonuclease was co-transfected into T. gondii parasites with a PCR product encoding a TEV-HA epitope tag flanked by 50 bp of sequence homologous to the regions immediately up- and down-stream of the TgMPPα stop codon. The homologous repair pathway of the parasite mediates integration of the PCR product into the TgMPPα locus. Forward and reverse primers were used to screen parasite clones for successful integration of the TEV-HA tag at the target site, yielding a 274 bp product in the native locus and a 414 bp product in the modified locus. (B) PCR screening analysis using genomic DNA extracted from putative TgMPPα-TEV-HA parasites (clones 1–6). Clones 1–4 and 6 yielded PCR products that indicated that these clones had been successfully modified. Genomic DNA extracted from wild type (WT) parasites was used as a control. (C) Diagram depicting the 3’ replacement strategy to generate TEV-HA-tagged TgCox2a. A sgRNA was designed to target near the stop codon of TgCox2a. A plasmid containing the sgRNA and GFP-tagged Cas9 endonuclease was co-transfected into T. gondii parasites with a PCR product encoding a TEV-HA epitope tag flanked by 50 bp of sequence homologous to the regions immediately up- and down-stream of the TgCox2a stop codon. Forward and reverse primers were used to screen parasite clones for successful integration of the TEV-HA tag at the target site, yielding a 260 bp product in the native locus and a 400 bp product in the modified locus. (D) PCR screening analysis using genomic DNA extracted from putative TgCox2a-TEV-HA parasites (clones 1–6). All 6 clones yielded PCR products that indicated that these clones had been successfully modified. Genomic DNA extracted from wild type (WT) parasites was used as a cont [file ppat.1009211.s001.tif]

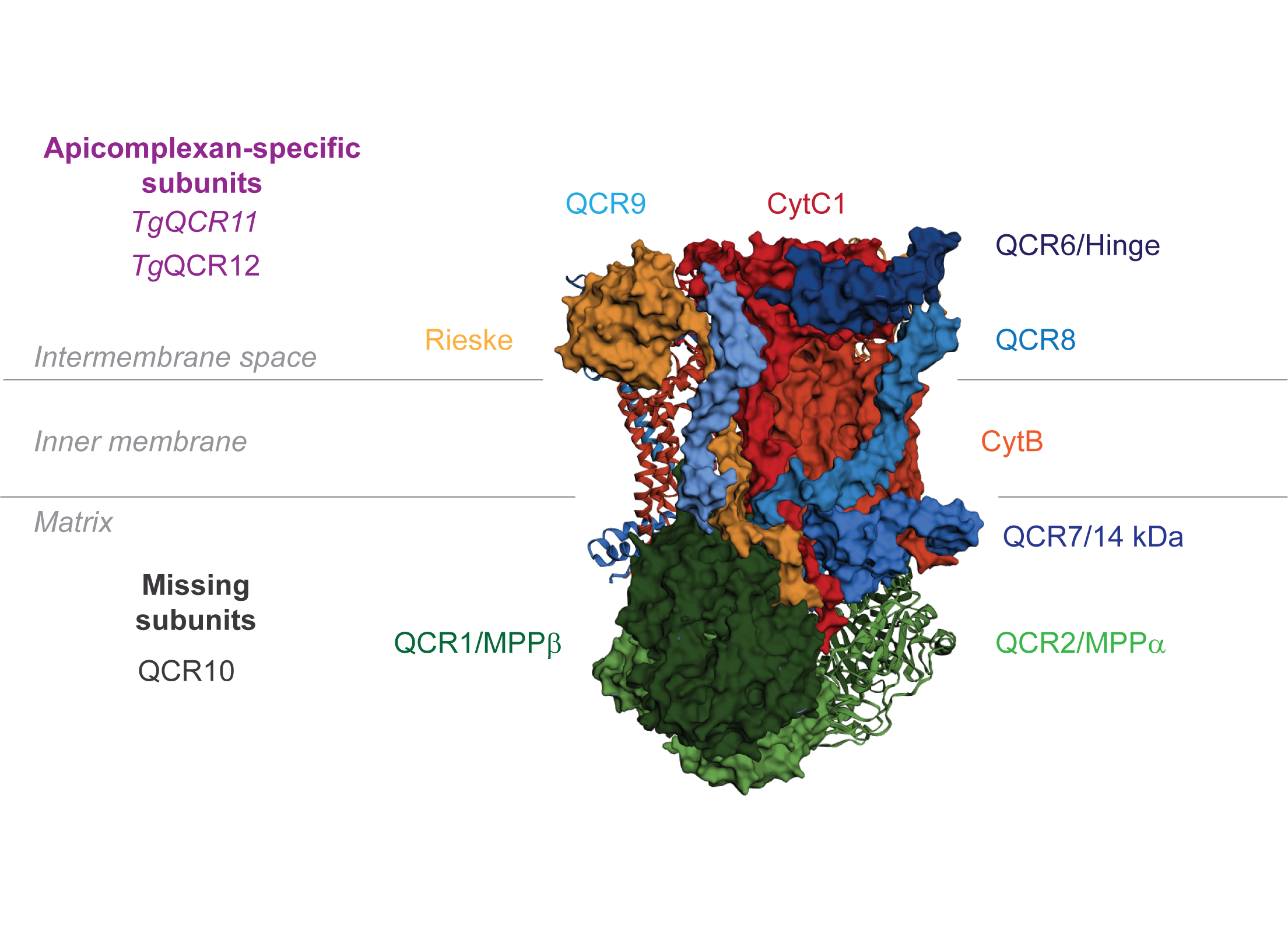

Supplement: S2 Fig — The yeast Complex III dimer structure (PDB: 3CX5) was imported into the EzMol program [64] and subunits were coloured as indicated, with one monomer of each subunit shown as surface display and the other as ribbons. No homolog of yeast QCR10 was detected in T. gondii (black), and no homologs of the T. gondii proteins TgQCR11 and TgQCR12 (purple) were found in yeast. (TIF) [file ppat.1009211.s002.tif]

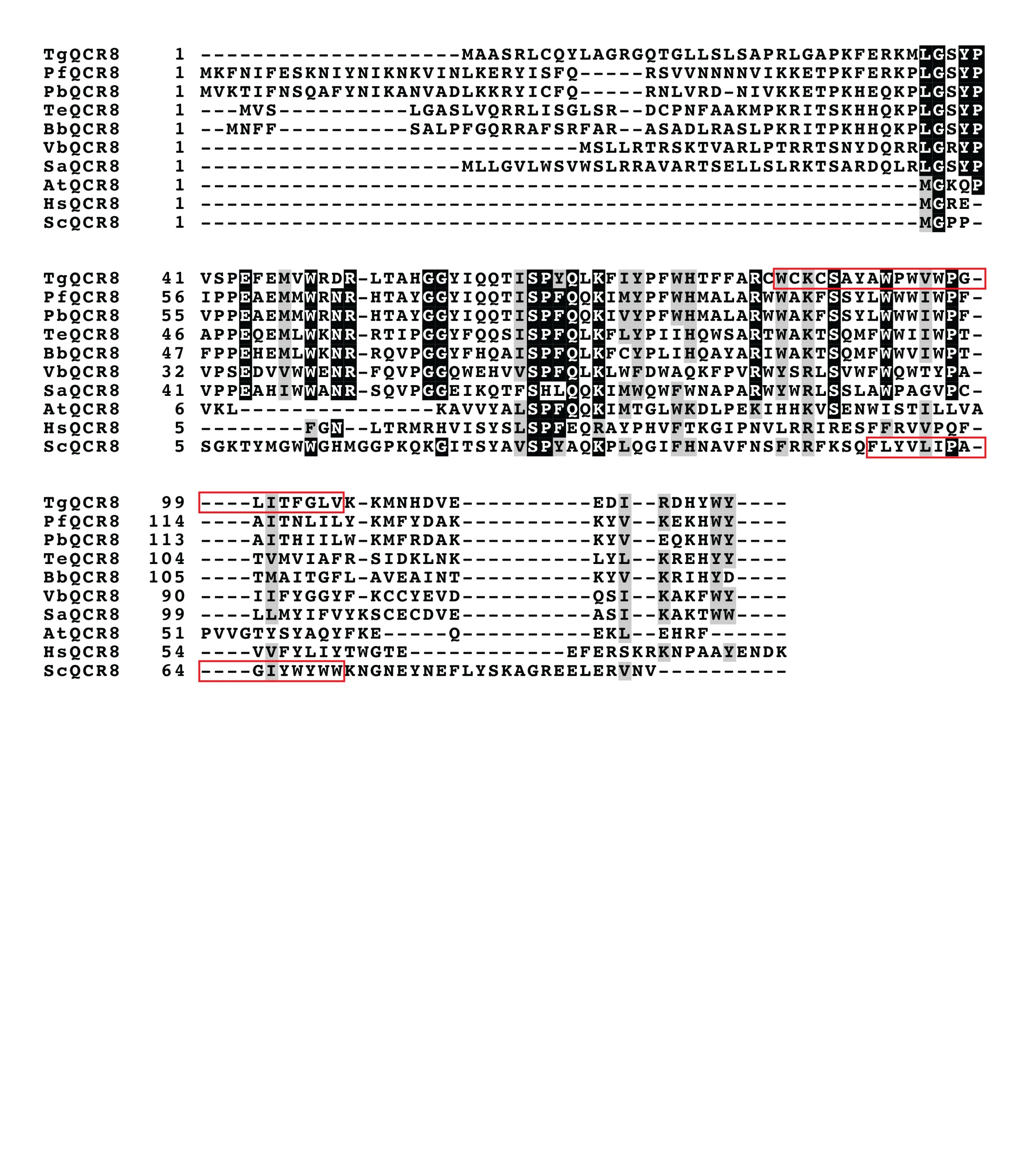

Supplement: S3 Fig — Alignment of QCR8 homologs from Toxoplasma gondii (TgQCR8; TGME49_214250), Plasmodium falciparum (PfQCR8; PF3D7_0306000), P. berghei (PbQCR8; PBANKA_0404400), Theileria equi (TeQCR8; BEWA_031210), Babesia bovis (BbQCR8; BBOV_IV004300), Vitrella brassicaformis (VbQCR8; Vbra_14054), Symbiodinium microadriaticum (SaQCR8; Smic7304), Arabidopsis thaliana (AtQCR8; NP_196156), Homo sapiens (HsQCR8; NP_055217) and Saccharomyces cerevisiae (ScQCR8; NP_012369). Dark shading indicates amino acid identity in ≥70% of the sequences, and light shading indicates amino acid similarity in ≥70% of the sequences. The positions of predicted transmembrane domains in TgQCR8 (TMPred prediction) and ScQCR8 (TMHMM prediction) are indicated by red boxes. (TIF) [file ppat.1009211.s003.tif]

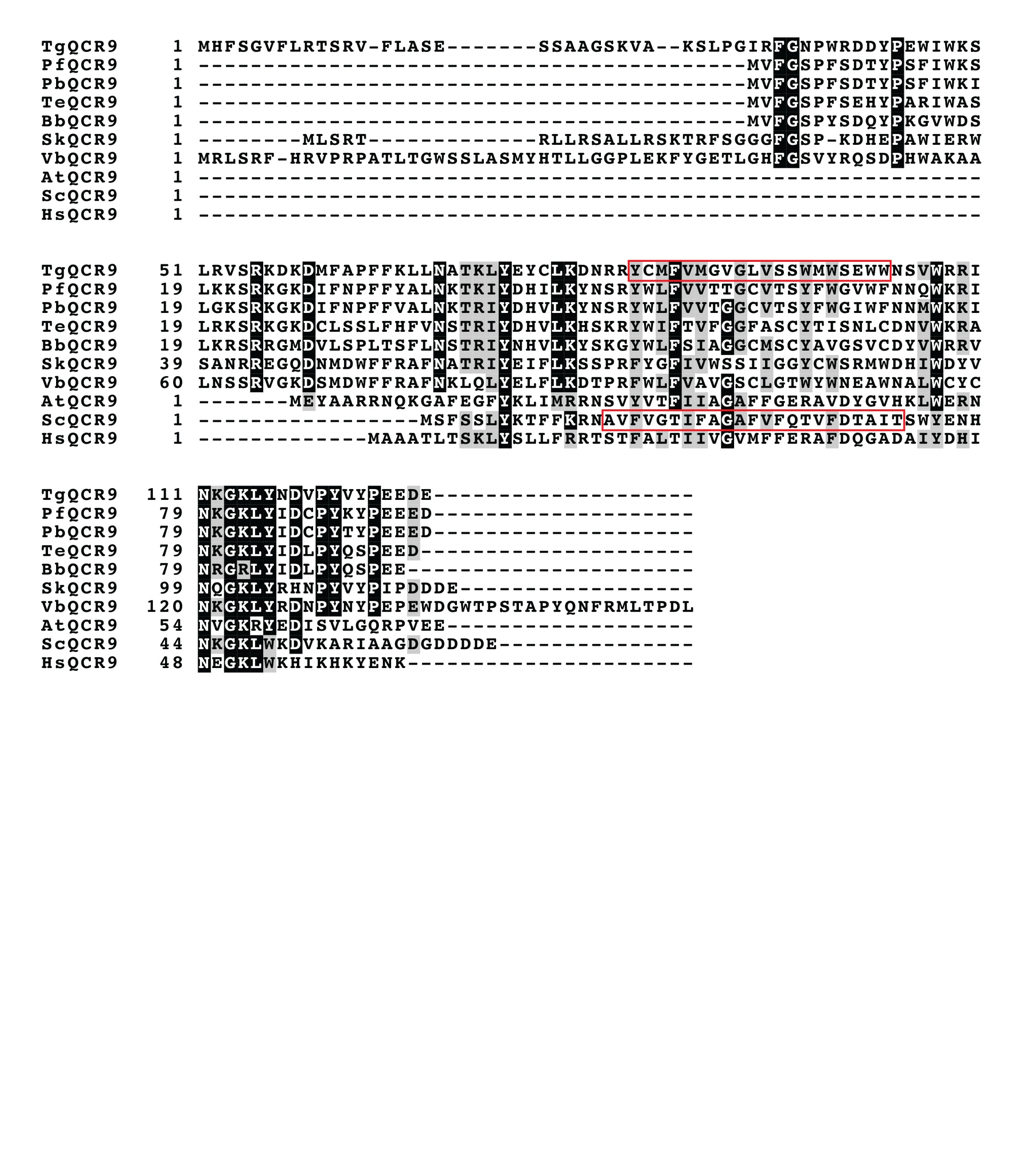

Supplement: S4 Fig — Alignment of QCR9 homologs from T. gondii (TgQCR9; TGME49_201880), P. falciparum (PfQCR9; PF3D7_0622600), P. berghei (PbQCR9; PBANKA_1121500), T. equi (TeQCR9; BEWA_007140), B. bovis (BbQCR9; BBOV_III007050), V. brassicaformis (VbQCR9; Vbra_943), Symbiodinium kawagutii (SkQCR9; Skav217368), Arabidopsis thaliana (AtQCR9; NP_190841), Homo sapiens (HsQCR9; NP_037519) and Saccharomyces cerevisiae (ScQCR9; NP_011699). Dark shading indicates amino acid identity in ≥70% of the sequences, and light shading indicates amino acid similarity in ≥70% of the sequences. The positions of predicted transmembrane domains in TgQCR9 (TMPred prediction) and ScQCR9 (TMHMM prediction) are indicated by red boxes. (TIF) [file ppat.1009211.s004.tif]

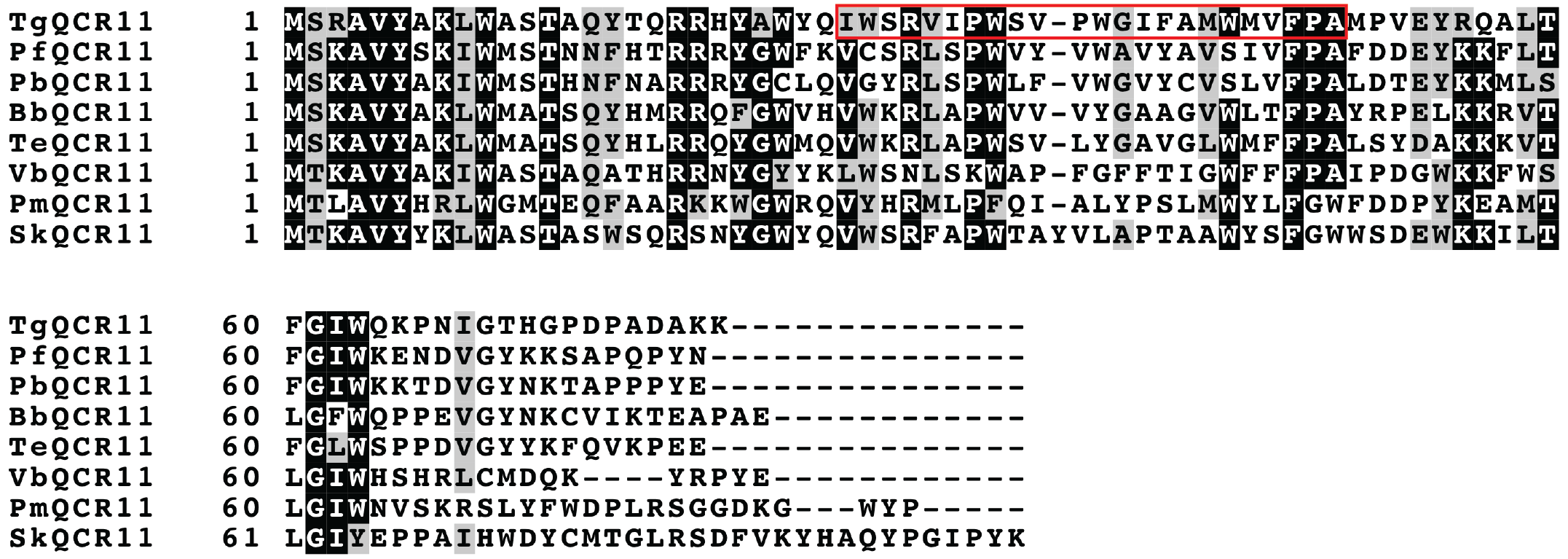

Supplement: S5 Fig — Alignment of QCR11 homologs from T. gondii (TgQCR11; TGME49_214250), P. falciparum (PfQCR11; PF3D7_0722700), P. berghei (PbQCR11; PBANKA_0620200), B. bovis (BbQCR11; BBOV_IV004900), T. equi (TeQCR11; BEWA_032020), V. brassicaformis (VbQCR11; Vbra_12339), Perkinsum marinus (PmQCR11; XP_002780203), and S. kawagutii (SkQCR11; Skav223196). Dark shading indicates amino acid identity in >70% of the sequences, and light shading indicates amino acid similarity in >70% of the sequences. The position of predicted transmembrane domains in TgQCR11 (TMHMM prediction) is indicated by a red box. (TIF) [file ppat.1009211.s005.tif]

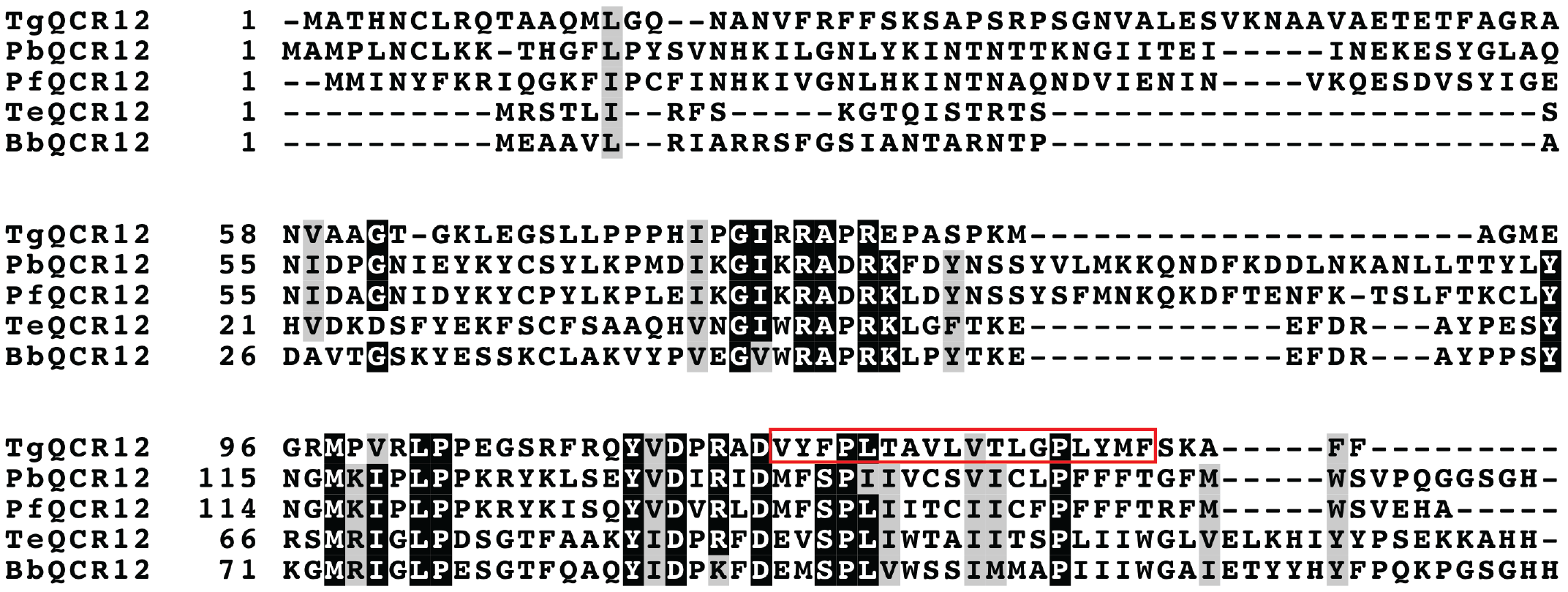

Supplement: S6 Fig — Alignment of QCR12 homologs from T. gondii (TgQCR12; TGME49_207170), P. berghei (PbQCR12; PBANKA_1341100), P. falciparum (PfQCR12; PF3D7_1326000), T. equi (TeQCR12; BEWA_021660), and B. bovis (BbQCR12; BBOV_III005260). Dark shading indicates amino acid identity in ≥80% of the sequences, and light shading indicates amino acid similarity in ≥80% of the sequences. The position of predicted transmembrane domains in TgQCR12 (TMHMM prediction) is indicated by a red box. (TIF) [file ppat.1009211.s006.tif]

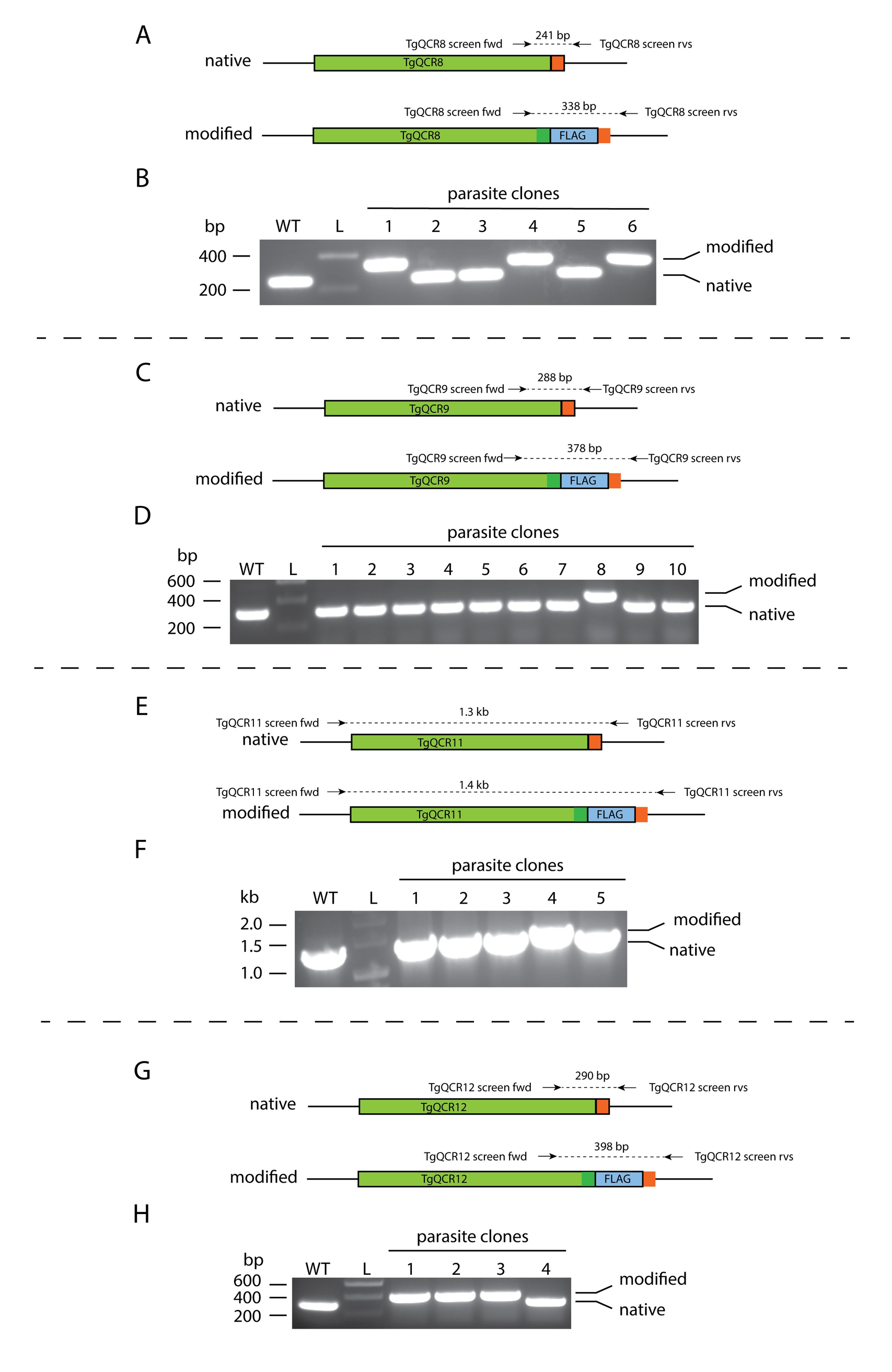

Supplement: S7 Fig — Diagrams depict the 3’ replacement strategy to FLAG-tag target genes. sgRNAs were designed to target the T. gondii genome near the stop codon of target genes. A plasmid containing the sgRNA and GFP-tagged Cas9 endonuclease was co-transfected into TgMPPα-HA T. gondii parasites with a PCR product encoding a FLAG epitope tag flanked by 50 bp of sequence homologous to the regions immediately up- and down-stream of the stop codon. Genomic DNA extracted from wild type (WT) parasites was used as a control in PCRs. (A) Forward and reverse primers were used to screen parasite clones for integration of the FLAG tag at the TgQCR8 locus, yielding a 241 bp product in the native locus and a 338 bp product in the modified locus. (B) PCR screening using genomic DNA extracted from putative TgQCR8-FLAG parasites (clones 1–6). Clones 1, 4 and 6 yielded PCR products that indicated that these clones had been successfully modified. (C) Forward and reverse primers were used to screen parasite clones for integration of the FLAG tag at the TgQCR9 locus, yielding a 288 bp product in the native locus and a 378 bp product in the modified locus. (D) PCR screening using genomic DNA extracted from putative TgQCR9-FLAG parasites (clones 1–10). Clone 8 yielded a PCR product that indicated it had been successfully modified. (E) Forward and reverse primers were used to screen parasite clones for integration of the FLAG tag at the TgQCR11 locus, yielding a 1.3 kb product in the native locus and a 1.4 kb product in the modified locus. (F) PCR screening using genomic DNA extracted from putative TgQCR11-FLAG parasites (clones 1–5). Clone 4 yielded a PCR product that indicated it had been successfully modified. (G) Forward and reverse primers were used to screen parasite clones for integration of the FLAG tag at the TgQCR12 locus, yielding a 290 bp product in the native locus and a 398 bp product in the modified locus. (H) PCR screening using genomic DNA extracted from putative TgQCR12-FLAG parasites (clo [file ppat.1009211.s007.tif]

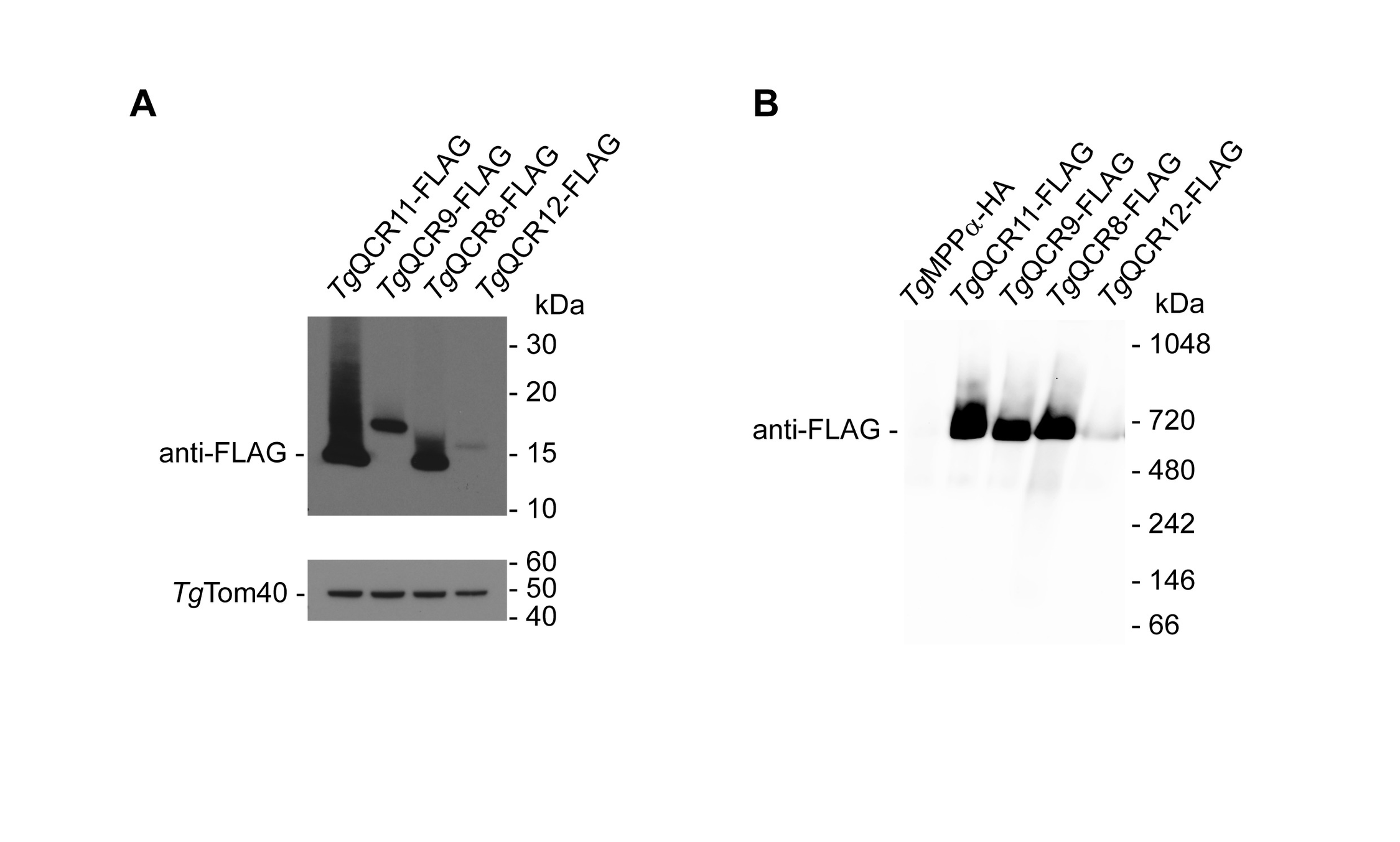

Supplement: S8 Fig — (A) Western blot of proteins extracted from TgMPPα-HA/TgQCR11-FLAG, TgMPPα-HA/TgQCR9-FLAG, TgMPPα-HA/TgQCR8-FLAG and TgMPPα-HA/TgQCR12-FLAG parasites, separated by SDS-PAGE, and detected with anti-FLAG and anti-Tom40 (loading control) antibodies. (B) Western blot of proteins extracted from TgMPPα-TEV-HA, TgMPPα-HA/TgQCR11-FLAG, TgMPPα-HA/TgQCR9-FLAG, TgMPPα-HA/TgQCR8-FLAG and TgMPPα-HA/TgQCR12-FLAG parasites, separated by BN-PAGE, and detected with anti-FLAG antibodies. (TIF) [file ppat.1009211.s008.tif]

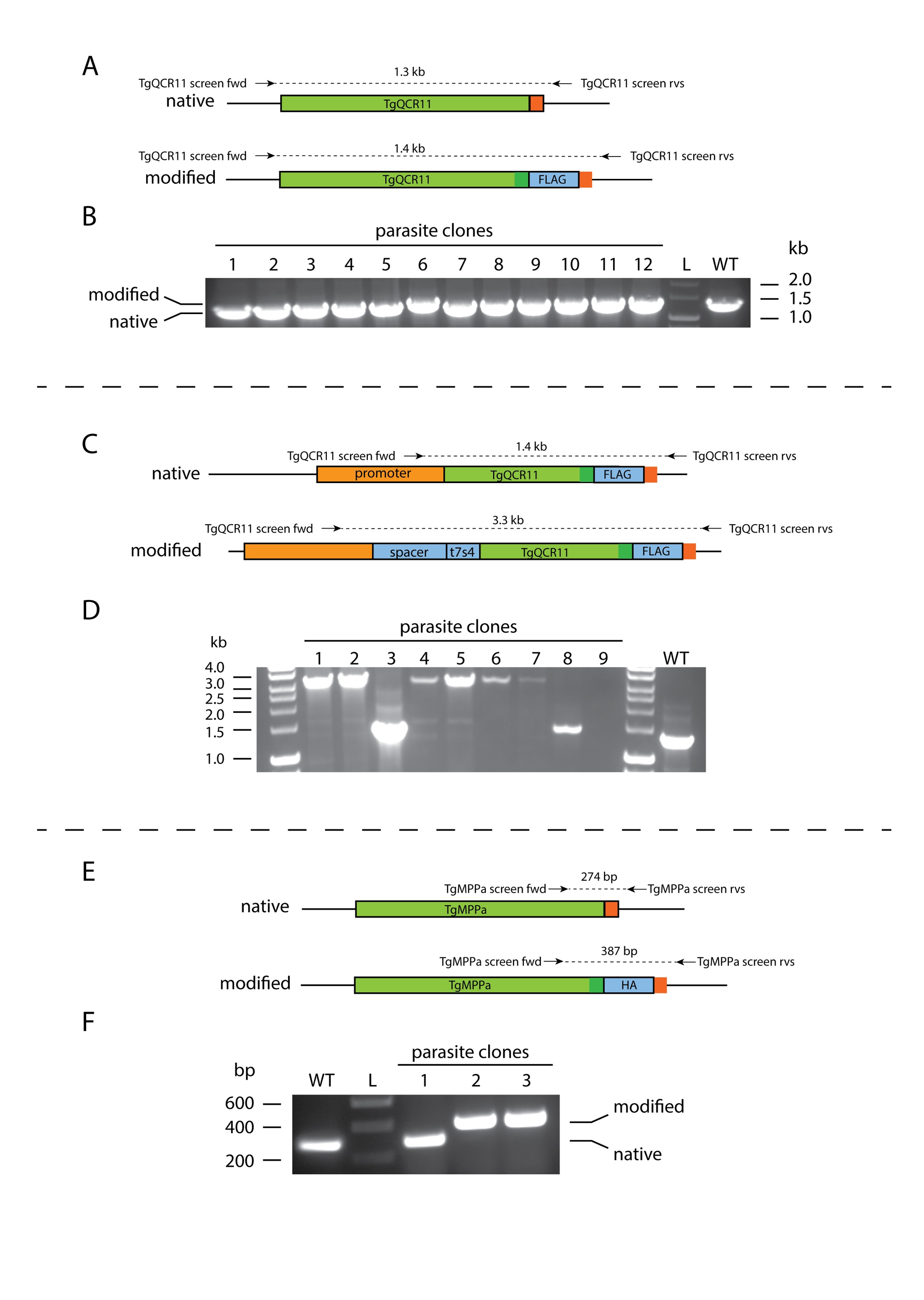

Supplement: S9 Fig — (A) Diagram depicting the 3’ replacement strategy to FLAG-tag TgQCR11. A sgRNA was designed to target the T. gondii genome near the stop codon of TgQCR11. A plasmid containing the sgRNA and GFP-tagged Cas9 endonuclease was co-transfected into T. gondii parasites with a PCR product encoding a FLAG epitope tag flanked by 50 bp of sequence homologous to the regions immediately up- and down-stream of the TgQCR11 stop codon. Forward and reverse primers were used to screen parasite clones for integration of the FLAG tag at the TgQCR11 locus, yielding a 1.3 kb product in the native locus and a 1.4 kb product in the modified locus. (B) PCR screening using genomic DNA extracted from putative TgQCR11-FLAG parasites (clones 1–12). Clone 6 yielded a PCR product that indicated it had been successfully modified. Genomic DNA extracted from wild type (WT) parasites was used as a control. (C) Diagram depicting the promoter replacement strategy to generate ATc-regulated TgQCR11. A sgRNA was designed to target the T. gondii genome near the start codon of TgQCR11. A plasmid containing the sgRNA and GFP-tagged Cas9 endonuclease was co-transfected into T. gondii parasites with a PCR product encoding the ATc regulated ‘t7s4’ promoter, which contains 7 copies of the Tet operon and a Sag4 minimal promoter, flanked by 50 bp of sequence homologous to the regions immediately up- and down-stream of the TgQCR11 start codon. The PCR product also contain a ‘spacer’ region that separates the regulatable promoter from the native promoter of the TgQCR11 gene to enable sufficient regulation. Forward and reverse primers were used to screen parasite clones for successful integration of the regulatable promoter at the TgQCR11 locus, yielding a 1.4 kb product in the native, FLAG-tagged locus and a 3.3 kb product in the modified locus. (D) PCR screening using genomic DNA extracted from putative rTgQCR11-FLAG parasites (clones 1–9). Clones 1, 2, 4–7 yielded PCR products that indicated that these clones had [file ppat.1009211.s009.tif]

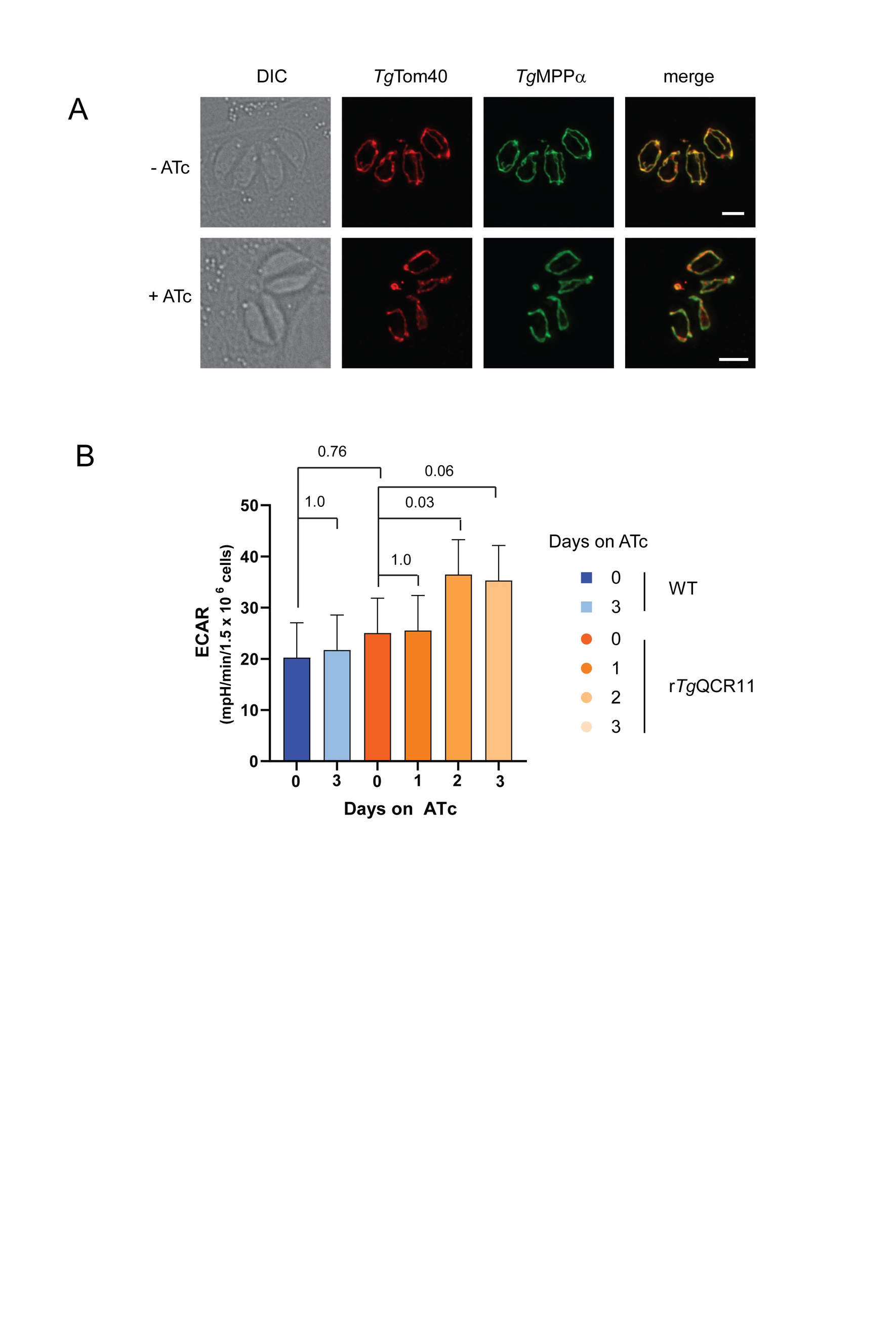

Supplement: S10 Fig — (A) Immunofluorescence assays assessing mitochondrial morphology in rTgQCR11-FLAG/TgMPPα-HA parasites grown in the absence of ATc (top) or in the presence of ATc for 3 days (bottom). The outer mitochondrial membrane was labelled using antibodies against TgTom40 (red), and the inner mitochondrial membrane was labelled using anti-HA antibodies to detect TgMPPα-HA. Images are representative of 100 four-cell vacuoles examined in 2 independent experiments; scale bar represents 2 μm. (B) Basal extracellular acidification rate (ECAR) of WT parasites grown in the absence of ATc or in the presence of ATc for 3 days (blue), and rTgQCR11-FLAG/TgMPPα-HA parasites grown in the absence of ATc or in the presence of ATc for 1–3 days (orange). A linear mixed-effects model was fitted to the data and values depict the least squares mean ± 95% CI of three independent experiments. ANOVA followed by Tukey’s multiple pairwise comparisons test was performed, with relevant p values shown. (TIF) [file ppat.1009211.s010.tif]

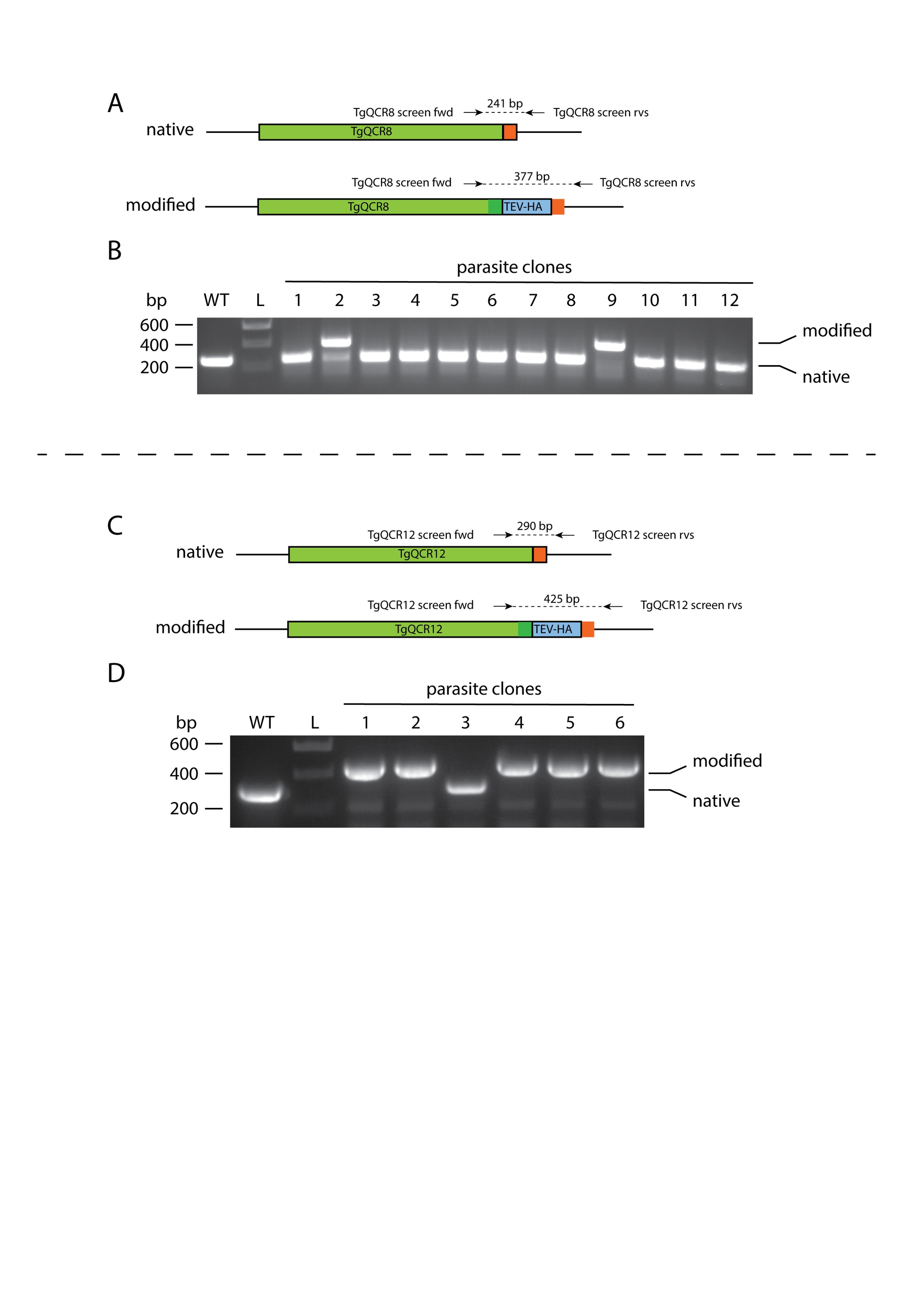

Supplement: S11 Fig — Diagrams depict the 3’ replacement strategy to TEV-HA-tag target genes. sgRNAs were designed to target the T. gondii genome near the stop codon of target genes. A plasmid containing the sgRNA and GFP-tagged Cas9 endonuclease was co-transfected into rTgQCR11-FLAG T. gondii parasites with a PCR product encoding a TEV-HA epitope tag flanked by 50 bp of sequence homologous to the regions immediately up- and down-stream of the stop codon. Genomic DNA extracted from wild type (WT) parasites was used as a control in PCRs. (A) Forward and reverse primers were used to screen parasite clones for integration of the TEV-HA tag at the TgQCR8 locus, yielding a 241 bp product in the native locus and a 377 bp product in the modified locus. (B) PCR screening using genomic DNA extracted from putative rTgQCR11-FLAG/TgQCR8-TEV-HA parasites (clones 1–12). Clone 9 yielded PCR products that indicated it had been successfully modified. (C) Forward and reverse primers were used to screen parasite clones for integration of the TEV-HA tag at the TgQCR12 locus, yielding a 290 bp product in the native locus and a 425 bp product in the modified locus. (D) PCR screening using genomic DNA extracted from putative rTgQCR11-FLAG/TgQCR12-TEV-HA parasites (clones 1–6). Clones 1–2 and 4–6 yielded PCR products that indicated that they had been successfully modified. (TIF) [file ppat.1009211.s011.tif]

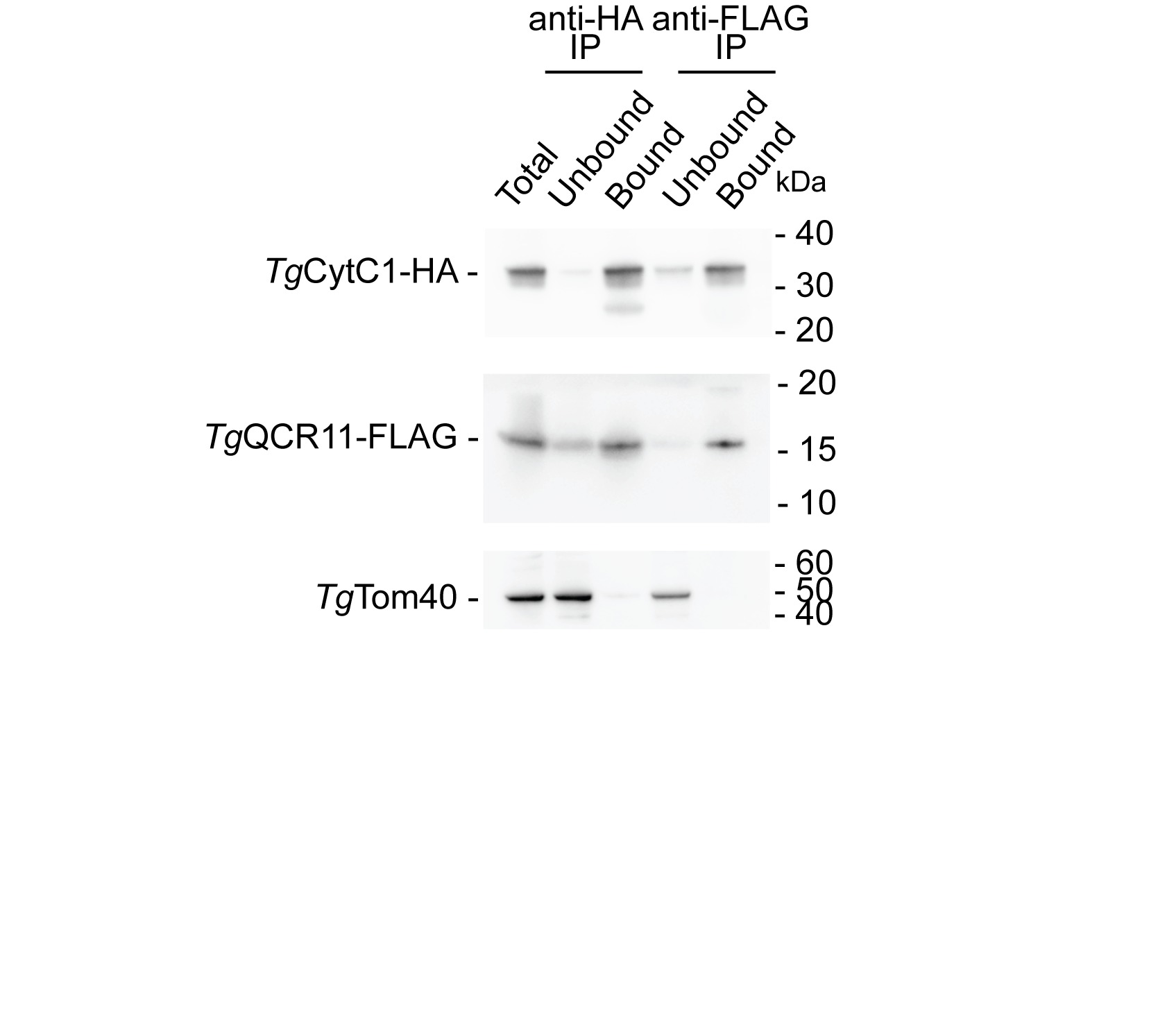

Supplement: S12 Fig — Western blots of proteins extracted from rTgQCR11-FLAG/TgCytC1-HA parasites, and subjected to immunoprecipitation using anti-HA (anti-HA IP) or anti-FLAG (anti-FLAG IP) antibody-coupled beads. Extracts include samples before immunoprecipitation (Total), samples that did not bind to the anti-HA or anti-FLAG beads (Unbound), and samples that bound to the anti-HA or anti-FLAG beads (Bound). Samples were separated by SDS-PAGE, and probed with anti-HA antibodies to detect TgCytC1-HA, anti-FLAG to detect TgQCR11, and anti-TgTom40 as a control to detect an unrelated mitochondrial protein. (TIF) [file ppat.1009211.s012.tif]

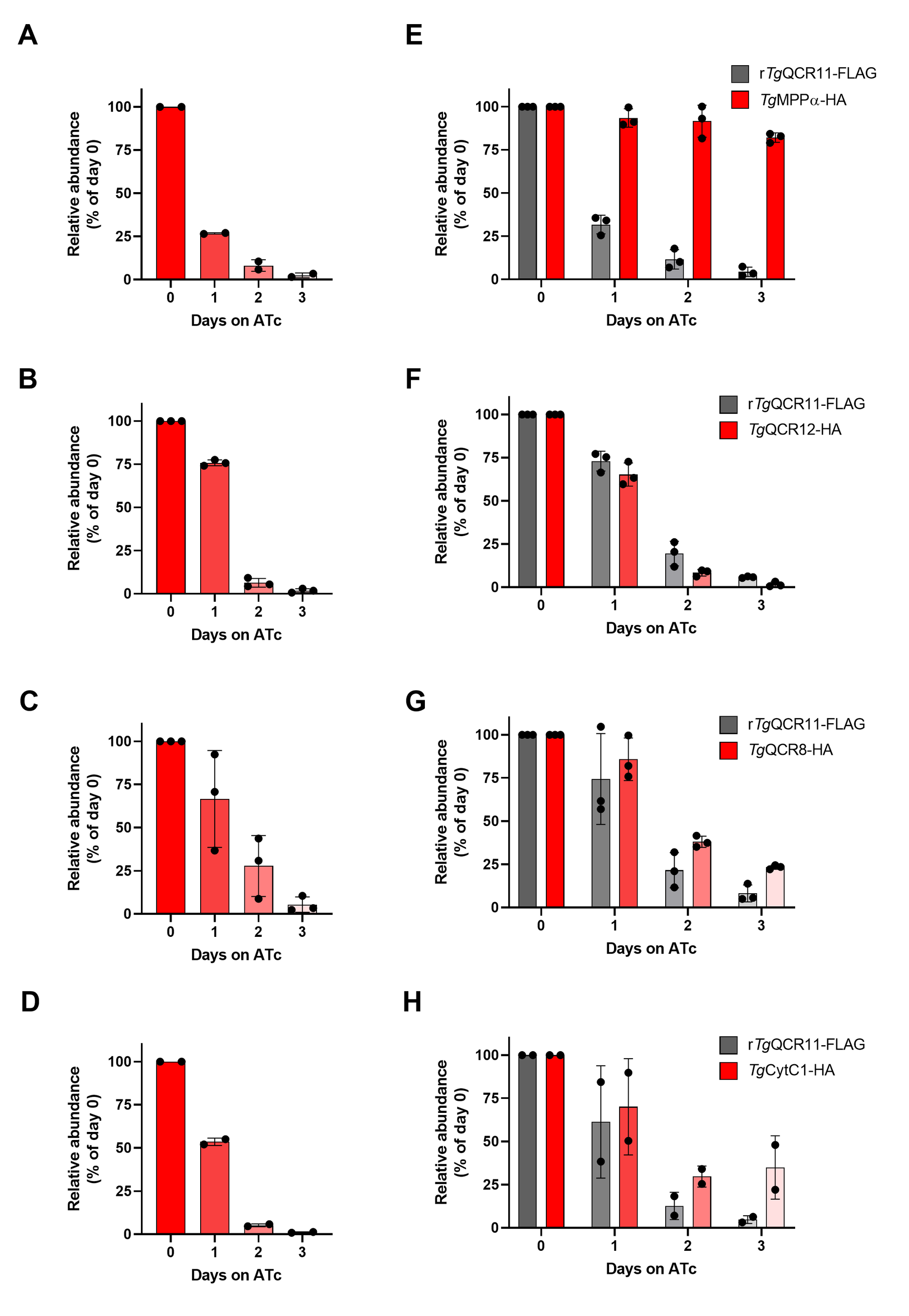

Supplement: S13 Fig — (A-D) Quantification of BN-PAGE western blots from Fig 6. (A) rTgQCR11-FLAG/TgMPPα-HA, (B) rTgQCR11-FLAG/TgQCR12-TEV-HA, (C) rTgQCR11-FLAG/TgQCR8-TEV-HA and (D) rTgQCR11-FLAG/TgCytC1-HA parasites were grown in the absence of ATc or in the presence of ATc for 1–3 days. Band intensities were normalized to the matched SDS-PAGE TgTom40 control and expressed as a percent of the day zero control. Columns represent the mean ± SD of at least 2 independent experiments, with individual values depicted. (E-H) Quantification of SDS-PAGE western blots from Fig 6. (E) rTgQCR11-FLAG/TgMPPα-HA, (F) rTgQCR11-FLAG/TgQCR12-TEV-HA, (G) rTgQCR11-FLAG/TgQCR8-TEV-HA and (H) rTgQCR11-FLAG/TgCytC1-HA parasites were grown in the absence of ATc or in the presence of ATc for 1–3 days. Band intensities were normalized relative to the TgTom40 control and expressed as a percent of the day zero control. Columns represent the mean ± SD of at least 2 independent experiments, with individual values depicted. rTgQCR11-FLAG is shown in gray tones and HA-tagged proteins are shown in red tones. (TIF) [file ppat.1009211.s013.tif]
