# Supplementary material for: Divergent features of the coenzyme Q:cytochrome c oxidoreductase complex in Toxoplasma gondii parasites
Source: PLoS Pathog. 2021 Feb 1;17(2):e1009211. doi: 10.1371/journal.ppat.1009211 (PMC7877769; doi:10.1371/journal.ppat.1009211)
Supplement: S2 Table — (PDF) [file ppat.1009211.s015.pdf]

| Oligonucleotide name | Oligonucleotide sequence (5’ to 3’) |
|---|---|
| MPPα 3’ rep CRISPR fwd | GCTTCACTTGCCGACGCCCGGTTTTAGAGCTAGAAATA GCAAG |
| Universal CRISPR rvs | ACTTGACATCCCCATTTAC |
| MPPα tag fwd | CGCACTACGAGGAGGTACGCGCTGCTCTCCGAGCAGCG GGCGTCGGCAAGGGTGGAGGTAGCGGTGGTGGAAG |
| MPPα tag rvs | ATGCAGCTTTCTTCGTTTCCGAGACCTTTCCAATTCTCT CTGCGCCCTGCGCTTCTGTGGGCGGTTATCAGG |
| Cox2a tag fwd | GACAGTGGTACTGGATCTACGAAGTCGAGTCGCCTGTT GACGACGAaGAGGGTGGAGGTAGCGGTGGTGGAAG |
| Cox2a tag rvs | CTGCCCATTCAACGCTCGGACAGCCGTCCTTTAGGAAA CGCATAGGAAGCGCTTCTGTGGGCGGTTATCAGG |
| MPPα screen fwd | TTTCTTTTTCGCTGTCCGATA |
| MPPα screen rvs | GTAGACACGTTCCTTCCTCTCG |
| Cox2a screen fwd | CTCTTGTACATGCTCGACGAAG |
| Cox2a screen rvs | AACGACTGTGATTCCAAAACCT |
| QCR8 3’ rep CRISPR fwd | TCGCAGGTATTAAGCGTCGTGTTTTAGAGCTAGAAATA GCAAG |
| QCR9 3’ rep CRISPR fwd | CCGGAGGAGGATGAGTAAACGTTTTAGAGCTAGAAAT AGCAAG |
| QCR11 3’ rep CRISPR fwd | CACTGTCTATTTCTTTGCGTGTTTTAGAGCTAGAAATAG CAAG |
| QCR12 3’ rep CRISPR fwd | AAGCTGGTTCTACAGTGCCGGTTTTAGAGCTAGAAATA GCAAG |
| QCR8 tag fwd | GAACTGGAAGAGAAGTGCGTCCTTTTTCTGGGTGTTTC GCTCGCAGGTATGGTGGAGGTAGCGGTGGTGGAAG |
| QCR8 tag rvs | CAGGGTTTCTCTCCCGCAAAGAGGCGAATCTGGACCGG CAAACATCGTTGGCTTCTGTGGGCGGTTATCAGG |
| QCR9 tag fwd | AACAGAAACTCTACAATGATGTCCCGTACGTCTATCCG GAGGAGGATGAGGGTGGAGGTAGCGGTGGTGGAAG |
| QCR9 tag rvs | GCCACGGGTGAGTGGAACCTTGCAGCTTCAGATTCGTG TTTGCATAGCAGGCTTCTGTGGGCGGTTATCAGG |
| QCR11 tag fwd | AAAAACCGAACATTGGCACTCACGGACCGGATCCTGCt GACGCAAAGAAAGGTGGAGGTAGCGGTGGTGGAAG |
| QCR11 tag rvs | GAGTTTGGCGACCGTGTTGTTGAGCCGTACTTCCCAAT GAAATGCTCTCGGCTTCTGTGGGCGGTTATCAGG |
| QCR12 tag fwd | CTGTTTTGGTGACGCTGGGGCCTCTCTACATGTTCTCCA AAGCCTTCTTCGGTGGAGGTAGCGGTGGTGGAAG |
| QCR12 tag rvs | TCTCGCGGCTTTCTTGGAGTTCGCGCCCCAGAGGAGAG ACTCGCACCACGGCTTCTGTGGGCGGTTATCAGG |
| QCR8 screen fwd | GTCTTCAGGGTCTTCTGTTGCT |
| QCR8 screen rvs | CTTCCGTTTTACGAGCTCAAGT |
| QCR9 screen fwd | CGTTTTCACACACACTACCCAT |
| QCR9 screen rvs | TGACTTGTGTTGCAGAGTAGGC |
| QCR11 screen fwd | TTTTTATCTATTCTGGGCCTGC |
| QCR11 screen rvs | CCCATACCTCACTGGTTTCTGT |

| | |
|---|---|
| QCR12 screen fwd | CGGACGTTTACTTTCCTCTCAC |
| QCR12 screen rvs | TGAAACAGTGTCCAGAGACGAC |
| QCR11 5’ CRISPR fwd | GGACATTCTGGCTCCGGCAGGTTTTAGAGCTAGAAATAGCAAG |
| QCR11 pro rep fwd | CGAGTTTTTTTCTGCCGAACAGGCGGTTATTCTCAAGGTAATTTCTCCCAGGTTGCAGGCTCCTTCTTCGG |
| QCR11 pro rep rvs | GTGTATTGAGCCGTGCTGGCCCAGAGCTTCGCGTAGACCGCGCGGGACATtttGGTTGAAGACAGACGAAAGCAGTTG |
| QCR11 comp fwd | CTAGAGATCTAAAATGTCCCGAGCGGTCTACGC |
| Universal Ty1 rvs | CTAGCCCGGGGCTTCTGTGGGCGGTTATCAGG |
| -TEV-HA gBlock | GGTGGAGGTAGCGGTGGTGGAAGTGAAAATCTGTACTTCCAGGGAGGTACCTACCCGTACGACGTCCCGGACTACGCTGGCTATCCCTATGATGTGCCCGATTATGCGTATCCTTACGATGTTCCAGATTATGCCTGATAACCGCCCACAGAAGC |
| FLAG gBlock | GTGGAGGTAGCGGTGGTGGAAGTGACTACAAAGACCATGACGGTGATTATAAAGATCATGACATCGATTACAAGGATGACGATGACAAGTAGTCCTGATAACCGCCCACAGAAGC |
| HA gBlock | GGTGGAGGTAGCGGTGGTGGAAGTTACCCGTACGACGTCCCGGACTACGCTGGCTATCCCTATGATGTGCCCGATTATGCGTATCCTTACGATGTTCCAGATTATGCCTGATAACCGCCCACAGAAGC |
| QCR11-Ty1 gBlock | ATGTCCCGAGCGGTCTACGCGAAGCTCTGGGCCAGCACGGCTCAATACACACAACGCAGACATTATGCGTGGTACCAAATCTGGTCGCGCGTGATTCCCTGGTCCGTGCCTTGGGGCATCTTCGCTATGTGGATGGTGTTCCCCGCCATGCCAGTTGAGTATCGTCAGGCGCTGACTTTCGGCATTTGGCAAAAACCGAACATTGGCACTCACGGACCGGATCCTGCCGACGCAAAGAAAGGTGGAGGTAGCGGTGGTGGAAGTGAGGTGCATACCAATCAAGACCCTTTGGATGAAGTCCATACCAATCAAGATCCTTTGGACGAGGTCCATACGAACCAGGACCCCTTGGACGGGGCCTGATAACCGCCCACAGAAGC |
