# Supplementary material for: Divergent features of the coenzyme Q:cytochrome c oxidoreductase complex in Toxoplasma gondii parasites
Source: PLoS Pathog. 2021 Feb 1;17(2):e1009211. doi: 10.1371/journal.ppat.1009211 (PMC7877769; doi:10.1371/journal.ppat.1009211)
Supplement: Attachment — Submitted filename: Response_to_reviewers.pdf [file ppat.1009211.s016.pdf]

Response to editor's comments

The reviewers are enthusiastic about the work both from a fundamental point of view as well as for its potential to define novel targets for therapeutic intervention. However, they identified some issues that will have to be addressed prior to consideration for publication. These include the need to discuss further some of your findings in the broader context of the literature (Rev #2 points 2 and 3).

To address Reviewer 2 point 2, we have added text to the discussion (lines 393-401) about the roles of yeast supernumerary subunits in Complex III function/integrity. We have also added broader context about the mode of action of Complex III inhibitors (targeting the Q_o and Q_i sites within cytochrome *b*) to the introduction (lines 75-77) to address Reviewer 2 point 3.

In addition, at least two main points will need to be address experimentally. Cyt c1 should be included as bait for IP/MS studies to consolidate and further validate the composition of the complex III (Rev #1 point 1).

We have HA epitope tagged *Tg*CytC1 in the regulatable *Tg*QCR11 (previously called *Tg*ApiQCR10) strain. We demonstrate a) that *Tg*CytC1 co-immunoprecipitates with *Tg*QCR11 (S12 Fig), b) that *Tg*CytC1 is part of a ~675 kDa complex (Fig. 6D), the same mass as the components of Complex III that we verify elsewhere in the manuscript, and c) that knockdown of *Tg*QCR11 decreases the abundance of the *Tg*CytC1 protein (Fig. 6H) and depletes the ~675 kDa *Tg*CytC1-containing complex (Fig. 6D). Together these data provide compelling evidence that *Tg*QCR11 is part of the same complex as *Tg*CytC1 (and by extension Complex III). We did not repeat the proteomic studies with *Tg*CytC1, since we believe that, collectively, the data we present provide more direct evidence that *Tg*CytC1 and *Tg*QCR11 are components of Complex III.

The possible existence of super-complexes should be explored under variable detergent concentration and if they exist assess the influence of QCR10 and QCR11 (Rev#2 point 1)

We have repeated blue native-PAGE studies of the *Tg*MPPα complex in several detergents, including digitonin, a detergent that has been used in studies from yeast, mammals and plants to solubilise intact super-complexes. We did not see any notable changes in the mass of the major *Tg*MPPα complex when solubilised in digitonin (Fig 1A), suggesting that, in the conditions we tested, super-complexes are not observed. Our data do not rule out the possibility that super-complexes exist in apicomplexans, but given all of the candidate Complex III components we examine in our study are in a complex of ~675 kDa, which differs in mass to Complex IV (PMID: 30204084, a common partner to Complex III in super-complexes), we can be confident that our study is examining Complex III in isolation. We would like to point out (as we also do below in response to comments from Reviewer 2) that our study is intended to examine the novel components of Complex III, and we believe that investigating the possible existence of super-complexes is beyond the scope of this study.

Response to reviewer comments

Major Comments

Reviewer #1:

1. MPP has been identified as a stable member of complex III in other organisms, but its interactions with complex III have not previously been studied in Apicomplexa. It seems odd then that the authors chose MPP as bait for identifying the protein components of complex III, rather than one of the main complex III components such as cyt c1, especially as the authors recently published a tagged cyt c1 line (ref. 25). In this regard, inclusion of cyt c1 as bait for IP/MS studies and as positive control for reciprocal IP studies in Fig. 3 would be a strong complement to the MPP data to triangulate complex III composition and tighten the conclusion that the identified 675 kDa complex indeed reflects complex III.

The choice of *Tg*MPPα as bait for the complex was an historical one, because we had an existing parasite strain in which *Tg*MPPα had been epitope tagged (PMID: 27458014) that we used in preliminary proteomic analyses. The data from Figure 1 establish that the major complex in which *Tg*MPPα resides is a ~675 kDa complex, which is a similar mass to the other candidate proteins that we identify in the complex (*Tg*QCR8, 9, 11 and 12; Fig 3A), and that *Tg*MPPα co-purifies with these proteins in co-immunoprecipitation experiments (Fig. 3B-E). We also demonstrate that *Tg*MPPα co-purifies with canonical components of Complex III using proteomic approaches (Fig. 1C-D). Additionally, we have now epitope tagged *Tg*CytC1 and demonstrate that this catalytic subunit of Complex III a) is part of a complex of the same mass as the other candidate Complex III components (Fig. 6D), b) co-purifies with *Tg*QCR11 by co-immunoprecipitation (S12 Fig), and c) that loss of *Tg*QCR11 leads to a depletion of both the *Tg*CytC1 complex and the *Tg*CytC1 protein (Fig. 6D, H). We believe that these data 'triangulate' Complex III composition, showing that *Tg*CytC1, *Tg*MPPα and newly identified components of the complex (e.g. *Tg*QCR11) are all part of the same 675 kDa complex.

2. Is complex IV the optimal negative control to use for IP/MS experiments? Super-complex formation between complexes III and IV occurs in other organisms. Although unstudied in Apicomplexa, any stable interactions between these complexes in *T. gondii* could lead to false negatives in the proteomic analysis of complex III components. Tom40 was used as a negative control in Fig. 3- why not use it as the negative control for IP/MS experiments? Conversely, why wasn't complex IV used as negative control in Fig. 3?

The overarching aim of our study was to identify Complex III components in *T. gondii*. On this basis, we believe that Complex IV is an optimal control for a couple of reasons. a) Using Complex IV as a negative control enables subtraction of any proteins that might be present in both complexes (e.g. proteins that mediate the formation of super-complexes in these parasites, if they exist). b) Complex IV is in the same membrane as Complex III (in contrast to *Tg*Tom40, which is in the outer membrane). If our purification of Complex III and IV simultaneously purify bulk inner membrane material, this material will be 'subtracted' in the proteomic analysis depicted in Fig1C-D. Regarding why Complex IV wasn't used as a negative control in Fig. 3, we have not generated a strain where both a Complex III and a Complex IV component are tagged, so are unable to do the experiment the reviewer suggests. We note, however, that the proteomic analysis in our study (Fig. 1C) identified all the same Complex IV components we identified in a previous study (PMID: 30204084), wherein we used *Tg*Tom40 as a negative control. This implies that *Tg*Tom40 and Complex IV are equally suited as negative controls for protein/proteomic analysis of Complex III.

3. Fig. 3A lacks an appropriate negative control. The authors should ideally show an anti-FLAG blot of a BN-PAGE with the parental MPP-HA only line to rule out antibody cross-reactivity.

We now include these data as S8B Fig. We observe minimal cross-reactivity with the anti-FLAG antibody.

Reviewer #2:

1. Investigations on ETC complexes from multiple organisms have shown the complexes assemble into very large super-complexes, sometimes referred to respirasomes. Cryo-EM structures of some of them have been solved (see e.g. PMID:27654917, PMID:27912063 and PMID:30598554). Supernumerary subunits seem to be critical for the assembly of these super-complexes. Authors here did not detect super-complexes in blue native (BN) gel electrophoresis, which could likely be due to the solubilization conditions they used; respirasomes are sensitive to detergent concentrations used. Clearly, proteomic analyses of the complexes require harsher detergent solubilization to avoid artefactual associations, but this could also eliminate associations that are significant. While it is possible that Toxoplasma ETC may not form super-complexes, this would be unusual. Authors should be encouraged to carry out BN gel electrophoresis under low to high detergent concentrations to assess the presence or absence of super-complexes. In addition, effects of knockdown of QCR10 and QCR11 on potential super-complexes should be assessed.

We have performed additional experiments that indicate that solubilisation of parasite proteins in 1% (w/v) digitonin, a detergent that solubilises intact super-complexes in other organisms, does not change the mass of the major *Tg*MPPα-containing complex (i.e. Complex III; Fig. 1A). We also show that the mass of Complex III when solubilised in 1% (v/v) Triton X-100 is different to the previously observed mass of Complex IV, a common partner of Complex III in super-complexes, in this detergent (~600 kDa; PMID: 30204084). Furthermore, proteomic analysis of the *Tg*MPPα and *Tg*Cox2a (a canonical component of Complex IV) complexes in 1% (w/v) dodecyl-maltoside identifies different subunits (Fig. 1C-D). These experiments indicate that, under the conditions of our protein extractions, we are examining Complexes III and IV is isolation, and that there are no observable super-complexes.

We cannot rule out the possibility that super-complexes exist in *T. gondii*. The purpose of our study was not to examine their existence, but rather to identify the protein composition of the isolated Complex III in these parasites. The experiments that we have undertaken show that proteins like *Tg*QCR11 and *Tg*QCR12 (previously termed *Tg*ApiQCR10 and *Tg*ApiQCR11, respectively) are bona fide components of Complex III in these parasites. Specifically, both of these proteins are part of a ~675 kDa complex (Fig. 3A), both co-purify with *Tg*MPPα (Fig. 1C,D and Fig 3B,E), *Tg*QCR11 also co-purifies with the catalytic Complex III subunit *Tg*CytC1 (S12 Fig), knockdown of *Tg*QCR11 leads to breakdown of the ~675 kDa Complex III and decreased abundance of numerous Complex III proteins including *Tg*QCR12 (Fig. 6), and, finally, knockdown of *Tg*QCR11 leads to a selective defect in the activity of Complex III in mediating parasite respiration (Fig. 5B, D).

2. Unlike the mammalian ETC, apicomplexans (just like the budding yeasts) lack Complex I but have a single subunit NADH dehydrogenase. This leads to a different arrangement of supercomplexes in the yeast. Proteins Rcf1 and Rcf2 are critical for the formation of CIII and CIV supercomplex in the yeast (PMID:22310663), and Rcf2 was recently shown to be part of the supercomplex by cryo-EM (PMID:32291341). These 2 yeast proteins are unique and not seen in other organisms. Authors should interpret their findings of unique QCR proteins in myxozoans in light of these and other results showing the role of such supernumerary subunits in ETC assembly and functions.

As noted in our previous response, our intention was not to characterise super-complexes or assembly factors for Complex III/super-complexes. Although examining the possible existence of super-complexes in these parasites is interesting, we believe this goes beyond the scope of our study. As also noted in our previous response, we provide numerous lines of evidence that the proteins we identify are bona fide components of Complex III, and that QCR11 has a specific role in Complex III function and/or assembly.

The reviewer suggests that we interpret our finding of unique QCR proteins in myzozoans in terms of literature about the yeast proteins Rcf1 and Rcf2. Rcf1 is actually not unique to yeast and has homologs in plants and animals (HIGD2A in humans), and more recent literature suggests that Rcf1 functions an assembly factor for the Cox3 subunit of Complex IV, rather than being directly required for formation of super-complexes or Complex III function (PMID: 30683696; PMID: 32317297). Similarly, while Rcf2 is indeed restricted to fungi, the cryo-EM structure of the yeast super-complex shows Rcf2 to be associated with Complex IV rather than Complex III and situated away from the interface between Complexes III and IV (PMID: 32291341). In support of this, Rcf2 knockout yeast show no defect in either super-complex formation or Complex III function (PMID: 30683696). For these reasons, we would not consider Rcf1/2 to be supernumerary subunits of Complex III and therefore do not discuss them in the manuscript.

We have included further discussion about the roles of supernumerary subunits in Complex III of yeast in complex function/integrity to lines 393-401: "In other eukaryotes such as yeast, the supernumerary subunits of Complex III contribute to complex assembly and/or stability [41, 42]. For example, yeast QCR9 (which is homologous to *Tg*QCR9) is important for maintaining integrity of Complex III [37, 43, 44], and yeast QCR10 (for which we identified no homolog in *T. gondii*) appears to have a role in stabilising the catalytic Rieske subunit in the complex [45]. Supernumerary subunits have also been proposed to contribute to the assembly of so-called "supercomplexes" between Complex III and other respiratory complexes [46, 47]. For example, the yeast Complex III subunits Cor1 (the homologue of *Tg*MPPβ) and QCR8 (the homolog of *Tg*QCR8) interact with Complex IV within these supercomplexes [46, 47]."

3. Authors advance their findings to suggest that they could form the basis for antiparasitic drug discovery. Selective toxicity of anti-parasitic drugs against CIII arises from the structural differences between the Qo and Qi sites within the cytochrome b encoded by the parasite and their hot mitochondrial genome, something that was noted 27 years ago (PMID:8459834). Of the numerous CIII inhibitors known, essentially all work through inhibition of either Qo or Qi site of the enzyme. It would be important for the authors to tame their argument and present it in the context of the vast literature on CIII inhibitors and their mode of action.

We have added a sentence to the introduction (lines 75-77) to provide more context to the Q_o and Q_i sites of Complex III being the target of anti-parasitic drugs: "The mechanism of action of Complex III inhibitors has been studied extensively, with most exploiting structural differences between the Q_o and/or Q_i sites of Complex III in parasites and host species to selectively target parasites [5-7]".

We believe that there is therapeutic value in understanding the entire protein composition of a drug target like Complex III, given that proteins unique to apicomplexans may influence the accessibility or efficacy of inhibitors against Complex III. Previous studies in *T. gondii* identified an atovaquone resistant mutant that did not have mutations in *Tg*CytB (PMID: 8331476; PMID: 10802314), implying that mechanisms of resistance beyond mutations in the Q_o and Q_i sites of cytochrome *b* may exist – it is conceivable that this may include mutations in some of the other subunits of the complex. Studying Complex III in more depth also enables a greater understanding of the effects of Complex III inhibitors on parasite physiology. In the

revised manuscript, we provide genetic and chemical data that indicate parasite viability is not affected upon short term impairment of Complex III (see response to Reviewer 1, comment 9).

Minor Comments

Reviewer #1:

4. A Scheme summarizing canonical subunits and membrane topology of complex III would be helpful in the introduction, especially to set the stage for what compoments may be conserved or divergent in Toxoplasma and also to suggest possible roles for the novel subunits identified herein.

We have now included a diagram depicting the structure of Complex III from yeast (S2 Fig), highlighting the components for which we identify homologues in the *T. gondii* complex. We also note which subunits lack homologues in *T. gondii* (yeast QCR10) as well as which proteins are novel in *T. gondii* (*Tg*QCR11 and *Tg*QCR12). We refer to this figure at relevant points in the text.

5. It is confusing to state that TGGT1_214250 lacks sequence homology to known proteins but refer to it as "TgApiQCR10". QCR10 is a known complex III sub-unit in yeast (https://www.yeastgenome.org/locus/S000003529).

We now refer to *Tg*ApiQCR10 as *Tg*QCR11, and *Tg*ApiQCR11 as *Tg*QCR12. This nomenclature was decided upon in consultation with MacLean and colleagues, who have undertaken a complementary analysis of Complex III in *T. gondii* (MacLean et al. BioRxiv, 2020; https://doi.org/10.1101/2020.08.17.252163).

6. Lines 160-161: The HHPRED analysis probes sequence not structural similarity. No structures have been determined for the T. gondii proteins so it is impossible to evaluate structural similarity. Rather, the HMM analysis at HHPRED identifies remote sequence similarity to proteins whose structures have been deposited at the PDB as a basis for generating a homology model.

We have changed this sentence to now read "… we queried each protein against the Protein Data Bank (PBD) using HHPRED, a profile hidden Markov model search tool designed to identify homologous proteins with limited sequence similarity" (Lines 165-167). We have also modified the part of the discussion where we mention this, now stating that "Our analysis also identified two highly divergent supernumerary subunits, *Tg*QCR8 and *Tg*QCR9, which were only identifiable using the HHPRED search tool." (Line 372-374).

7. The authors should ideally give the e-value and/or probability scores returned by HHPRED for sequence similarity for TGGT1_227910 to yeast QCR8 and TGGT1_201880 to yeast QCR9.
We now mention the HHPRED e-values and probability scores for QCR8 and QCR9 in the text (lines 168-169).

8. Fig. 6D-F: I suggest that the authors quantify band intensity normalized to Tom40 to provide a more quantitative basis for conclusions about the effect of ApiQCR10 KD on stability of

QCR8, QCR11, and MPP. The current qualitative comparisons are difficult to evaluate, especially in Fig. 6F where ApiQCR10 KD appears weak.

We have now quantified band intensity normalised to *Tg*Tom40 for all blots in Fig 6 (presented in S13 Fig). When the relative abundance is plotted as a percent of the day zero sample, *Tg*QCR11 is knocked down by 92-96% by day 3 on ATc. We have also provided a more representative image for Fig 6F (now Fig 6G), since the image we included in the initial submission was overexposed.

9. For the experiment in Fig. S9B, how does the viability/recovery of parasites after ApiQCR10 KD compare to 24-48-hr treatment with atovaquone, which should phenocopy loss of complex III activity for on-target activity? Is viability of the KD parasites due to residual protein expression and trace complex III function or an ability to switch to glycolysis? Related, what does parasite viability despite QCR10 KD and loss of mitochondrial respiration suggest or imply about the efficacy of atovaquone or novel treatments that target ETC function?

We thank the reviewer for suggesting this interesting experiment. We grew parasites in the presence of atovaquone for 2 days before washing out the drug and allowing parasites to grow for a further 7 days. Our data indicate that parasites remain viable after 2 days treatment with atovaquone (Fig 4E), which phenocopies knockdown of *Tg*QCR11 (Fig 4D). In terms of how parasites might remain viable without a functional ETC - upon *Tg*QCR11 knockdown, parasites exhibit increased extracellular acidification rate (ECAR; Fig 4F and S10B Fig), which could indicate parasites remain viable via upregulation of other metabolic pathways such as glycolysis; however, this needs to be studied more directly before solid conclusions can be drawn.

In addition to describing these experiments in the results (Lines 257-264), we have added a paragraph to the discussion to consider what these data imply about the efficacy of atovaquone and other ETC inhibitors (lines 428-445): "We observed that parasites remained viable after knockdown of *Tg*QCR11 (Fig 4D), and that this was phenocopied by 2 day treatment with the Complex III inhibitor atovaquone (Fig 4E). These findings suggest that, at least in the short term and at the inhibitor concentration tested, parasites can survive but do not thrive in the absence of mitochondrial respiration. Precisely how parasites achieve this state remains uncertain, though the measurable increases in ECAR observed upon *Tg*QCR11 knockdown (Fig 4F; S10B Fig) suggests that parasites may respond to the impairment of Complex III by upregulating other metabolic pathways (such as glycolysis). Our earlier study of Complex IV demonstrated that parasites remain viable after loss of a key protein in this complex (TgApiCox25) [17], suggesting that parasite persistence is not specific to impairment of Complex III but is a general phenomenon when targeting the ETC. Determining the mechanism and duration of parasite persistence following the impairment of mitochondrial respiration has potentially important implications for therapeutics that target Complex III. Several clinical studies reported that 12-26% of patients experienced relapse of *T. gondii* infection after 1 year, and up to 75% after 6 years, following atovaquone therapy [48-50]. While the inability of atovaquone to eliminate *T. gondii* infection could be due to many factors, including the conversion of disease-causing tachyzoites to latent bradyzoite-containing tissue cysts [5], it is conceivable that the short-term persistence following ETC impairment may enable parasites to survive initial treatment until bradyzoite differentiation and/or atovaquone resistance arises."

10. It would be helpful if the authors can give the acrylamide % used for BN-PAGE experiments.

We now indicate the percent acrylamide using in both the SDS-PAGE and BN-PAGE gels in the methods section (Lines 527-529).

Reviewer #2:

1. Line 55: Clarify that electrons are passed from *reduced* CoQ to the *heme* within cytochrome b protein.

We now state that "… electrons from reduced CoQ are donated to a heme moiety on cytochrome *b* …" (Lines 52-53).

2. Lines 61 and 66: there are better references for these statements than #9 and #10.

We have now referenced a bovine complex IV structure paper at line 58 (PMID: 8638158) and the original chemiosmotic hypothesis paper at line 63 (PMID: 13771349).

3. Lines 75 and 76: Discovery of novel proteins in CIII does not necessarily open avenues for drug development. No examples are know for such inhibitors working on CIII.

In the referenced text, we state that "Therapeutically, the discovery of novel proteins in a critical pathway such as the ETC opens avenues for drug development." We are not claiming that the new proteins we identify are, necessarily, drug targets (although we also don't rule this out). Rather, as we mentioned in our response to major comment 3 from this reviewer, we are making the point that it is important to learn all that we can about drug targets, including their protein composition and their role in parasite physiology. We have changed the wording of this sentence: "Therapeutically, the discovery of novel proteins in a critical pathway such as the ETC highlights differences between parasites and their mammalian hosts that could open avenues for drug development" (Lines 72-74).

4. Line 139: The organelle in Cryptococcus parvum is termed a mitoplast and is not considered an authentic mitochondrion, reduced or otherwise.

We have changed the text to "contains a highly reduced mitochondrion (termed mitosome) that lacks Complex III" (line 142). We would like to point out that the common ancestor of apicomplexans had a 'true' mitochondrion that lost numerous functions in *Cryptosporidium parvum* (and several other *Cryptosporidium* species). Despite this, it retains numerous mitochondrial functions, including Fe-S synthesis and a reduced ETC.

5. QCR10 is appears to be expressed at a much greater level compared to other subunits, yet its signal in the BN gel appears to be stoichiometric. This could suggest additional functions associated with this protein. Authors should acknowledge this possibility.

QCR10 (now *Tg*QCR11) seems similar in abundance to *Tg*QCR8 by both BN and SDS-PAGE (S8 Fig), which in yeast/mammals has 1:1 stoichiometry with other *Tg*QCR proteins. We present evidence that *Tg*QCR11 is important for Complex III function (Fig 5). We also show that *Tg*MPPα immunoprecipitation co-purifies almost all *Tg*QCR11 (Fig 3B), suggesting that almost all of it is present in Complex III. While we can't rule out other functions of *Tg*QCR11, without better evidence we have chosen not to over-interpret this.

6. Authors should clearly state in the Results sections that OCR and ECAR measurements were done in isolated tachyzoites.

We now specify this in Lines 234 and 283.

Reviewer #3:

1. TgApiQCR11 levels are a lot lower than other complex III subunits (Sfig 7). The authors should consider that this protein is an assembly factor rather than a stoichiometric subunit. I understand that the protein appears to assemble into a complex that is similar in size to complex III, but since the lane in Fig. 3A is independent of the other samples, this cannot be clarified. It would therefore be appropriate to repeat the BN-PAGE blot with all samples on the same blot and show different exposures to capture the high molecular weight complex signal.

The blot presented in Fig 3A actually did had all samples on a single blot, which we cut and probed with different concentration of antibodies; we have added the following text to the Fig 3 legend to better explain this: "Images were obtained from a single membrane with lanes cut as indicated and probed with different concentration of antibodies" (Lines 897-898). To address the reviewer's comment, we have included an additional uncut BN-PAGE blot with all samples probed at a single antibody concentration and exposure (S8B Fig). As the reviewer rightly observes, both the SDS-PAGE and BN-PAGE results suggest that *Tg*ApiQCR11 (now *Tg*QCR12) is less abundant than the other Complex III subunits studied.

Although our data say nothing about the functional role of *Tg*QCR12 (e.g. whether it is a Complex III assembly factor), the co-immunoprecipitation data indicates that all of *Tg*QCR12 is in complex with *Tg*MPPα, and that *Tg*QCR12 pulls down a similar proportion of *Tg*MPPα as the other QCR proteins (Fig 3E). *Tg*QCR12 is also part of a similarly sized protein complex as other Complex III proteins (Fig 3A). Furthermore, knockdown of *Tg*QCR11 leads to a depletion of both the *Tg*QCR12 complex and the *Tg*QCR12 protein (Fig. 6B, F), indicating that the abundance of *Tg*QCR12 is dependent on *Tg*QCR11, which suggests that they are part of the same complex. Together, all of these data indicate that *Tg*QCR12 is a bona fide subunit of Complex III, although it may not exist in 1:1 stoichiometry with the other subunits. We have modified the discussion slightly to be clearer around this point (lines 366-368): "A caveat to this conclusion [that the sum of each subunit of the complex adds up to observed mass of the complex as a dimer] is that we observed different abundances in some Complex III subunits (S8 Fig), raising the possibility that not all subunits exist in a strict 1:1 stoichiometry per monomer."

2. The authors could also validate/assess whether complex III exists in a supercomplex as found in other organisms by using digitonin instead of DDM. The migration of the subunits with that larger form (especially TgApiQCR11) would help clarify that it is a bona fide complex III subunit.

As noted above in response to other reviewer comments, we did not observe evidence of supercomplexes in the conditions tested (including in 1% w/v digitonin; Fig 1A), though this does not rule out their existence in *T. gondii*. Therefore, while our data cannot inform the question of whether *Tg*QCR12 is part of a supercomplex, our data indicate that *Tg*QCR12 is a bona fide subunit of Complex III for the reasons listed in our previous response.
